# A Component-Based Leisure Activity Assessment for Adults with Autism Spectrum Disorder: A Preliminary Investigation

**DOI:** 10.1007/s40617-025-01071-y

**Published:** 2025-06-23

**Authors:** Robert W. Isenhower, Robert H. LaRue, Jenna L. Budge, James C. Maraventano

**Affiliations:** 1 Graduate School of Applied and Professional Psychology, Department of Applied Psychology, Douglass Developmental Disabilities Center, Rutgers University, 25 Gibbons Circle, New Brunswick, NJ 08901 USA; 2 Graduate School of Applied and Professional Psychology, Department of Applied Psychology, Rutgers Center for Adult Autism Services, Rutgers University, New Brunswick, NJ USA

**Keywords:** Adults, Choice, Concurrent operant, Leisure activities, Preference

## Abstract

Individuals with autism spectrum disorder (ASD) often have limited opportunities to participate in leisure activities, and behavior analysts need guidance in identifying activities their clients prefer. To support both groups, we present a user-friendly assessment that considers client preference and activity engagement when determining suitable leisure activities for individuals with ASD. Three adults with ASD who required significant support participated across three phases. During the first phase, concurrent operant arrangements were used to develop a preference profile for three leisure activity components: social interaction versus no interaction, electronic versus nonelectronic activities, and stationary activities versus those requiring movement. All participants showed clear preferences. The second phase used the resulting preference profile to assess engagement and the occurrence of problem behavior with leisure activities that matched or did not match their profile. Participants were more engaged with matched activities. Although problem behavior was rare, it occurred at lower rates with activities matched to the preference profile. The final phase assessed preference for matched versus unmatched leisure activities, with all participants preferring matched activities. These findings add to the literature by demonstrating an objective method for designing and evaluating new leisure experiences under controlled but naturalistic conditions. • The current study provides a new data-driven approach to identifying leisure preferences for adults with ASD. • The current study evaluates components of leisure activities to tailor leisure options effectively. • Leisure activities aligned with preferences result in higher engagement and fewer problem behaviors. • The assessment is adaptable for various populations, including transition planning and aging clients. • The current study fills a gap by offering an efficient means of evaluating preference for leisure activities.

We spend a significant part of our lives outside of work. Engaging in recreational and leisure activities allows us the opportunity to create an enjoyable and socially fulfilling structure in our daily routines. We may choose to fill our free time with activities such as watching television or streaming shows, playing video games, going to the gym, walking in nature, or participating in classes like yoga or dance. Although it is not possible to provide an exhaustive list, leisure involves activities that are freely chosen, give one pleasure, and are not directly work or school-related (Dattilo & Schleien, 1994). Reading a book for pleasure is an example of leisure, whereas reading for work or class is not. Similarly, cooking a favorite meal just because you enjoy it would qualify as leisure, while cooking to feed your family is more of a responsibility. Even playing on a voluntary work-sponsored softball team can be considered leisure, as it is not time spent doing the same tasks performed at work, even though it may involve colleagues or be organized by your employer. For all individuals, leisure is more than just a way to pass the time—it is essential to building a satisfying and well-rounded life (Manente et al., 2022).

Through leisure, individuals organize their free time in ways that can foster connection, creativity, and personal fulfillment. The benefits of engaging in leisure activities are numerous. Research shows that leisure can improve both physical and mental health (Caldwell & Gilbert, 1990), enhance mood and overall well-being (Han & Patterson, 2007), reduce stress and prevent burnout (Wolff et al., 2021), and even increase productivity (Cui et al., 2019). For individuals with autism spectrum disorder (ASD) and other related disabilities, research has demonstrated that leisure can reduce stress (Bishop-Fitzpatrick et al., 2018), reduce challenging behavior (Bremer et al., 2016), and improve overall quality of life (Rimmer et al., 2007).

Despite the numerous benefits, individuals with ASD have limited opportunities to participate in leisure activities and, as a result, are less likely to experience these positive effects. This may be partly because adult service providers often have significantly fewer resources, such as funding and staffing, to implement evidence-based approaches compared to those available for school-aged individuals with ASD (Gerhardt & Lainer, 2011). Where resources are limited, devoting time to creating effective adaptive leisure programs for adults with ASD is often seen as beneficial but not as critical as improving employment, communication, and self-care skills (Manente et al., 2022).

Dattilo and Schleien (1994) asserted that access to leisure is an essential human right. Service providers for individuals with ASD and other developmental disabilities should, therefore, provide significant support in these areas. Although the opportunity to engage in leisure activities is recognized as critical, they are rarely integrated systematically into individuals' lives. While formal preference assessments can help identify specific preferred items and activities, they offer limited insights into the structure of the leisure experience—such as whether the activity is interactive, where it takes place, and how it might best meet individual needs and interests. In short, behavior analysts need better guidance in identifying and predicting which leisure activities their clients will enjoy.

Many existing leisure and recreation research tools, such as the Leisure Satisfaction Scale (LSS; Beard & Ragheb, 1980), the Leisure Assessment Inventory (LAI; Hawkins et al., 1998), and the Children’s Leisure Assessment Scale (CLASS; Rosenblum et al., 2010), are based on indirect assessments like questionnaires and structured interviews. For example, the LSS evaluates individuals’ satisfaction with leisure in six categories: psychological, educational, social, physiological, relaxation, and aesthetic. The authors define satisfaction as the positive perceptions or feelings an individual forms through engagement in leisure activities. The LSS allows for a thorough, albeit indirect, understanding of individuals’ overall leisure satisfaction. Using the LSS, Stacey et al. (2019) found that adults with ASD reported lower levels of fulfillment related to leisure activities compared to their neurotypical peers. Stacey and colleagues also found that higher leisure satisfaction scores in adults with ASD correlated with fewer symptoms of depression, suggesting a positive relationship between meaningful leisure experiences and mental health. This gap in leisure satisfaction among adults with ASD suggests a need for more comprehensive approaches to support leisure with these individuals and promote a well-rounded quality of life.

While indirect assessments like the LSS can be helpful starting points, their results may not accurately gauge a person’s genuine interest in participating in an activity. These tools may be subject to biases, especially if they are completed by caregivers or others with differing perceptions of the individual’s preferences (Steege et al., 2019). Indirect assessments are also less practical for individuals with significant communication challenges due to their frequent use of self-report methods, such as questionnaires and interviews.

Numerous systematic methods for directly assessing individual preferences, including leisure activities, have been developed over the past 40 years. These stimulus preference assessments (SPAs) can be categorized on the basis of whether and how they constrain client access to each stimulus. On this basis, SPAs can be divided into limited access, free access, and response restriction (RR) methods. We briefly review each in turn.

In limited access methods, the individual is restricted in interacting with stimuli, usually by constraining the number of stimuli presented simultaneously or the time allowed to interact with each item. The most well-known of these assessments are named on the basis of the number of stimuli presented simultaneously, either one (e.g., single stimulus; Pace et al., 1985), two (e.g., paired stimulus; Fisher et al., 1992), or many (multiple stimulus without replacement [MSWO]; Deleon & Iwata, 1996). In these methods, data are usually collected on item selection and/or duration of engagement, and data across trials are summarized as the percentage of selection or time spent in engagement. A hierarchy of putative reinforcers can be constructed when directly comparing items, as in paired stimulus and MSWO assessments. These methods help identify preferred items and activities for individuals with limited verbal repertoires. Although researchers can examine limited types of engagement (e.g., Hagopian et al., 2001), these assessments generally do not provide deeper insights into how individuals might prefer to engage with the activities (e.g., dribbling a ball by themselves vs. playing catch with another person; bowling with a real ball in an alley vs. using a Nintendo Switch or iPad app).

In free access methods, individuals can interact with items without restrictions or forced choices (Roane et al., 1998). Duration of engagement with each stimulus is recorded to determine relative preference. Because stimuli are provided continuously, free access methods may reduce problem behavior associated with stimulus withdrawal (see also Kang et al., 2010). Unlike limited access assessments, free access assessments may offer greater insight into how individuals prefer to engage with items. However, a potential issue is that individuals may only engage with one item during the assessment, limiting the ability to gauge their preferences for the other items and how they might engage with them.

Hanley et al. (2003) introduced an RR assessment to address this limitation and evaluated leisure activity preferences in individuals with developmental disabilities. The RR assessment systematically limits access to highly preferred activities and measures how individuals reallocate their responses to remaining activities. By restricting access to the most preferred activities over successive sessions, the RR assessment allows for a more nuanced analysis of how individuals engage with various stimuli. This approach creates a clear preference hierarchy and enables observation of engagement with all activities, including those that were initially less preferred. Combining the strengths of both free- and limited-access methods, the RR assessment offers a more complete picture of an individual’s activity preferences.

While traditional SPAs typically focus on presenting one or more items or activities at a time to capture relative preferences, leisure activities often involve complex combinations of stimuli that may be differentially preferred when embedded within different contexts (e.g., greater opportunity for interaction; less movement required). With traditional assessment methods, evaluating all potentially preferred variations of leisure activities would take tremendous time and effort. Moreover, while SPAs are all valuable for identifying activities that might function as reinforcers, to varying degrees, they provide limited information about how individuals would prefer to engage with each activity, such as whether the activity is interactive or whether it requires movement.

LaRue et al. (2020) introduced a *component-based* approach to examining client preference and engagement in another complex skill domain—vocational tasks. Their assessment started by decomposing complex vocational tasks into distinct components or dimensions, like stationary vs. movement, interactive vs. noninteractive, and repetitive vs. complex. For example, a task like hanging clothing on hangers and arranging the clothing by size on racks requires the individual to stand and move, involves a fair amount of repetition, and offers limited opportunities for social interaction. This assessment tested components individually, and response allocation for the different comparisons was documented. The combination of preferred components created an individual client *profile*. The researchers then created new jobs that were aligned or misaligned with the preferences informed by each client’s profile. Jobs matched to these profiles yielded higher engagement (e.g., increased on-task behavior), reduced problem behavior, and were more preferred by participants compared to mismatched jobs. The individualized profile created by this analysis could be tested against various jobs and environments. Assessment outcomes (i.e., job matching) are improved because preferred components are related broadly to aspects of jobs that transcend specific professions and industries.

Given the utility of the component-based vocational assessment developed by LaRue et al. (2020), similar procedures could be used to design matched leisure activities. Such a leisure assessment model could not only help identify preferred stimuli and activities but also help design leisure experiences to match preferences (e.g., alone or with others, activities requiring movement or sedentary activities). Therefore, the primary objective of this study was to adapt the general procedures from LaRue et al. to pilot a direct assessment with three adults with ASD and high support needs to match them to new leisure activities they prefer and would likely engage with independently and spontaneously.

## Method

### Participants

Three male individuals enrolled in a university-based adult day program participated in the study. All participants were diagnosed with ASD and moderate to severe intellectual disability. The Vineland Adaptive Behavior Scales—Third Edition (Vineland-3; Sparrow et al., 2016) was administered to the parent(s) of each of the participants to assess their adaptive behavior.

Andrew was a 41-year-old man diagnosed with ASD and Fragile X syndrome. Standardized scores for his Vineland-3 placed him in the low (20–70) range for all three domains, Communication (20), Daily Living Skills (30), and Socialization (20), and the overall Adaptive Behavior Composite (24). His parents reported that he spoke using single words and short phrases and followed three-step instructions for familiar tasks. Though Andrew communicated vocally without interrupting, he did not start conversations with others by discussing their interests.

Jameson was a 36-year-old man diagnosed with ASD. Standardized scores for his Vineland-3 placed him in the low (20–70) range for all three domains, Communication (20), Daily Living Skills (20), and Socialization (20), and the overall Adaptive Behavior Composite (20). His parents reported that he spoke primarily using single words and followed instructions involving one action and one object. While Jameson could recognize emotions in others, he did not initiate conversations or typically try to make friends with peers.

Liam was a 39-year-old man diagnosed with ASD. Standardized scores for his Vineland-3 placed him in the low (20–70) range for all three domains, Communication (20), Daily Living Skills (24), and Socialization (20), and the overall Adaptive Behavior Composite (22). His parent reported that he spoke in complete sentences and followed two-step instructions. Liam did not typically engage in spontaneous or reciprocal conversation.

### Setting and Materials

Sessions occurred in a large conference room (approximately 5 m by 7 m). Masking tape divided the room in half. Identical tables (approximately 0.75 m by 2.75 m) were positioned along the wall on both sides of the room, and a chair was positioned in front of each table when appropriate. During the interaction condition, an experimenter sat in a second chair next to the table on one side of the room. Activities and materials were placed on the tables or the floor next to the tables, when appropriate, during relevant test conditions. Other materials included data sheets, pens/pencils, video cameras, and timers. Table 1 lists the leisure activities in Phase 1, and Table 2 lists leisure activities used in Phases 2 and 3. Activities in Phase 1 (Table 1) were selected from ones the clients had recently participated in at the day program, as observed by the experimenters and staff. For Phases 2 and 3 (Table 2), activities were selected on the basis of those experimenters and staff observed being engaged by *other* clients at the day program but not the clients themselves.

**Table 1:** Leisure activities used in Phase 1

Participant	Nonelectronic	Electronic	Stationary	Movement
Andrew	Cardboard puzzle	Puzzle on iPad	Coloring	Bouncing a kickball
	Drawing	Drawing on iPad	Writing	Dribbling a soccer ball
Jameson	Cardboard puzzle	Puzzle on iPad	Coloring	Bouncing a kickball
	Drawing	Drawing on iPad	Writing	Dribbling a soccer ball
Liam	Jenga	Jenga on iPad	Operation	Bowling
	Dominos	Dominos on iPad	Uno	Dribbling a soccer ball

**Table 2:** Matched and unmatched leisure activities used in Phases 2 and 3

Participant	Matched	Unmatched
Andrew	Fruit Ninja, seated, w/ staff interaction Temple Run, seated, w/ stall interaction	Playing basketball alone Bowling alone
Jameson	Fruit Ninja, seated, w/ staff interaction Temple Run, seated, w/ stall interaction	Playing basketball alone Bowling alone
Liam	Jeopardy on iPad, seated Bowling on iPad, seated	Frisbee with staff Basketball with peer

### Procedure

#### Phase 1: Leisure Component Preference Assessment

In the first phase of the investigation, a concurrent-operant assessment was used to determine the components of the leisure activities participants preferred. During the initial trial of each component, participants were provided 15-s of presession exposure to each option (e.g., interaction and no-interaction). The choice was provided while the participant stood at the center of the room on the tape line. If participants did not choose within 10-s, the participant was repositioned in the center of the room, presession access to choices was repeated, and the choice was presented again. Although rare, if participants continued to have difficulty choosing, then presession exposure was reintroduced in subsequent trials. The 2-min condition began when the participant moved to one side of the room. Participants could move freely from one side of the room to the other during the session. Trained observers monitored the time allocated to each side of the room. The side of the room on which each leisure activity was placed and the order of presession exposure was balanced across sessions. Each test was conducted at least four times for each participant or until a definitive pattern of preference, defined as one choice selected at least two-thirds (66.7%) of the time emerged.

All participants were given choices related to critical aspects of leisure activities. LaRue et al. (2020) identified interaction vs. no-interaction and stationary vs. movement. The third component, electronic vs. nonelectronic, was new to this analysis. In adding this component, we aimed to expand leisure activity options and reflect modern preferences, as electronic activities play a significant role in leisure.

##### Interaction vs. No-interaction

For this test, participants could choose between interacting with a staff member or sitting at a table alone for this test. Staff members ranged from novel individuals to those who frequently worked with the clients. The staff member selected for each session was based on convenience (i.e., the person already working with them). The experimenter began each session with verbal directions, “You can sit at this table by yourself, or you can sit at this table with [person’s name]. Pick one.” If the participant chose interaction, then the staff member would lead the participant in casual conversation. Conversations were not constrained in their content, and interactions ranged from generic comments (e.g., comments about clothing or the weather) to specific interests (e.g., the music of Tina Turner and Shania Twain). If the participant initiated a topic of conversation, the staff was instructed to follow their conversational lead. If the participants chose no interaction, then they would sit alone at the table without receiving attention.

##### Stationary vs. Movement

 For this test, participants could choose between an activity that required them to stand and frequently change positions (e.g., dribbling a basketball while standing) or to remain seated in a chair at the table (e.g., playing with a tennis ball while seated). The experimenter began each session with verbal directions, “You can sit and play [activity], or you can stand and play [activity]. Pick one.”

##### Electronic vs. Nonelectronic

 For this test, participants could choose between an activity displayed on an electronic device (e.g., a puzzle on the iPad) or a nonelectronic analog (e.g., a cardboard puzzle). Activities were equated in terms of task and difficulty. For example, if the nonelectronic condition used a 16-piece puzzle, the electronic condition used a 16-piece puzzle on the iPad. The experimenter began each session with verbal directions, “You can sit at this table and play with [activity] on the iPad, or you can sit at this table and play with the [activity]. Pick one.”

Phase 1 results were used to develop a leisure activity preference profile for each participant we applied to Phase 2. The profile was based on the time allocated across the different assessment conditions. For example, if the participant allocated most of their time to noninteractive, stationary, non-electronic activities, activities fitting that description *matched* the preference profile, and activities fitting the description of interactive, movement, and electronic were considered *unmatched*.

### Response Measurement and Interobserver Agreement

Graduate students and trained staff members collected data on the time allocated to each choice, defined as the number of seconds spent on either side of the divided room. A second independent observer collected data during 66.7% of sessions during Phase 1 of the investigation. Interobserver agreement (IOA) was calculated for the percentage of time allocated in each condition. The agreement for the time allocated was calculated by taking the total number of seconds from one observer and dividing it by the total number of seconds recorded by the second observer. IOA averaged 99.8% across all participants (range, 97.5–100%). IOA was high, in part, because participants did not often switch sides of the room after making their initial choice.

#### Phase 2: Analysis of Leisure Profile

In this phase, participants were exposed to two distinct types of leisure activities based on the results of Phase 1. First, we designed leisure activities that matched the client profile of the leisure preference assessment. We then designed unmatched activities using the opposite of the profile produced by the assessment. For example, if a participant’s leisure activities profile suggested that they preferred activities that involved low levels of social interaction, little movement, and an electronic interface, an activity that matched this profile (e.g., playing a basketball game alone on the iPad) would be compared to an activity with the opposite profile of high level of social interaction, required movement, and a nonelectronic interface (e.g., shooting basketball while conversing with a peer or staff member). Two matched and two unmatched activities were compared for each participant for Phase 2 (Table 2).

### Design, Response Measurement, and Interobserver Agreement

A multielement design was used to evaluate the differences in activity engagement across matched and unmatched activities. Sessions lasted 2 min. Matched and unmatched activities were evaluated at least three times each, and the specific activities used in each session were randomly selected from the list in Table 2.

Graduate students and trained staff members collected data on client engagement and frequency of problem behavior with each activity. Activity engagement was defined as the percentage of 10-s whole intervals oriented to the designated activity while using the materials how they were generally designed to be used. This definition was consistent across all three participants. Frequency data on the occurrence of problem behavior was collected and converted to responses per minute for each client. Operational definitions for each participant’s target behaviors are summarized next.

#### **Andrew**

Swearing was defined as any instance of profanity. Spitting was defined as the forceful expulsion of saliva from the mouth directed at another person, excluding instances where Andrew spat on the ground outdoors, in a trash can, or into a sink. Property disruption was defined as any instance or attempt to forcefully displace an object at least 6 in/15.24 cm from its original position without appropriate functional purpose or permission. Aggression was defined as an instance or attempt of forceful contact between any part of Andrew’s body and another individual, which included strikes with an open or closed hand, head butting, kicking, pushing, pinching, or biting. A previous functional analysis (FA) indicated that most topographies of Andrew’s behavior were maintained by automatic reinforcement. He had a behavior support plan (BSP) in place at the time of assessment and engaged infrequently in problem behavior during the day.

#### **Jameson**

Property disruption was defined as any instance or attempt of forceful displacement of an object 6 in/15.24 cm or greater from its original position without appropriate functional purpose or permission. Self-injury was defined as any instance or attempt of forceful contact between head and hand(s) or leg and hand(s) from a distance of at least 6 in/15.24 cm. Aggression was defined as any instance or attempt of forceful contact between any part of Jameson’s body and another individual, including strikes with an open or closed hand, head butting, scratching, or biting other individuals. Previous ABC data indicated that most topographies of Jameson’s behavior were maintained by automatic reinforcement. He had a BSP in place at the time of assessment and engaged infrequently in problem behavior during the day.

#### Liam

Shirt biting was defined as any instance in which Liam placed the fabric of his shirt or jacket into his mouth and bit down on it with his teeth. Self-injury was defined as any instance of forceful contact between a part of Liam’s body and another part of his body or object (e.g., hitting the table or hitting his leg). Aggression was defined as any attempt or forceful contact between any part of Liam’s body and another individual from 4 in/10.16 cm or greater, which included striking another with an open or closed fist, grabbing, and squeezing. Recent FA data indicated that Liam’s behavior was maintained by access to routines. He had no BSP in place at the time of assessment and engaged in moderate rates of problem behavior during the day.

A second independent observer collected data during 50% of sessions across all participants. Interval-by-interval IOA for activity engagement was calculated first by scoring observer data as agreements or disagreements for each 10-s interval. The number of intervals with agreements was divided by the total number of intervals and then converted to a percentage. IOA averaged 97.0% across all participants (91.7–100%). IOA for combined inappropriate behavior was calculated using exact agreement by dividing the number of agreements (a 10-s interval in which both observers scored the same frequency of problem behavior) by the number of agreements plus disagreements and converting the ratio to a percentage. IOA averaged 98.3% across all participants (range, 91.7–100%).

#### Phase 3: Preference for Matched and Unmatched Activities

A concurrent-operant assessment was implemented to determine participant preference for matched and unmatched leisure activities. Activities were placed on opposite sides of the room (with a staff member on the appropriate side for the interaction component) and were available concurrently. The side of the room on which each leisure activity was placed, and the order of presession exposure was balanced across trials. The same matched and unmatched activities from Phase 2 were used in Phase 3 (Table 2). Participants were asked to choose between the matched activity and the unmatched activity. If participants did not choose within 10-s, the participant was repositioned in the center of the room, and the choice was presented again. Trained observers monitored the choices made by the participants. The preference assessment consisted of at least four-choice trials.

### Response Measurement and Interobserver Agreement

Graduate students and trained staff members collected data on client activity selection. A second independent observer collected data during 41.7% of sessions. IOA was calculated by taking the total number of agreements (the same activity selected), dividing by the total number of agreements and disagreements, and converting the ratio to a percentage. IOA was 100% across all participants.

## Results

### Andrew

**Fig. 1:**
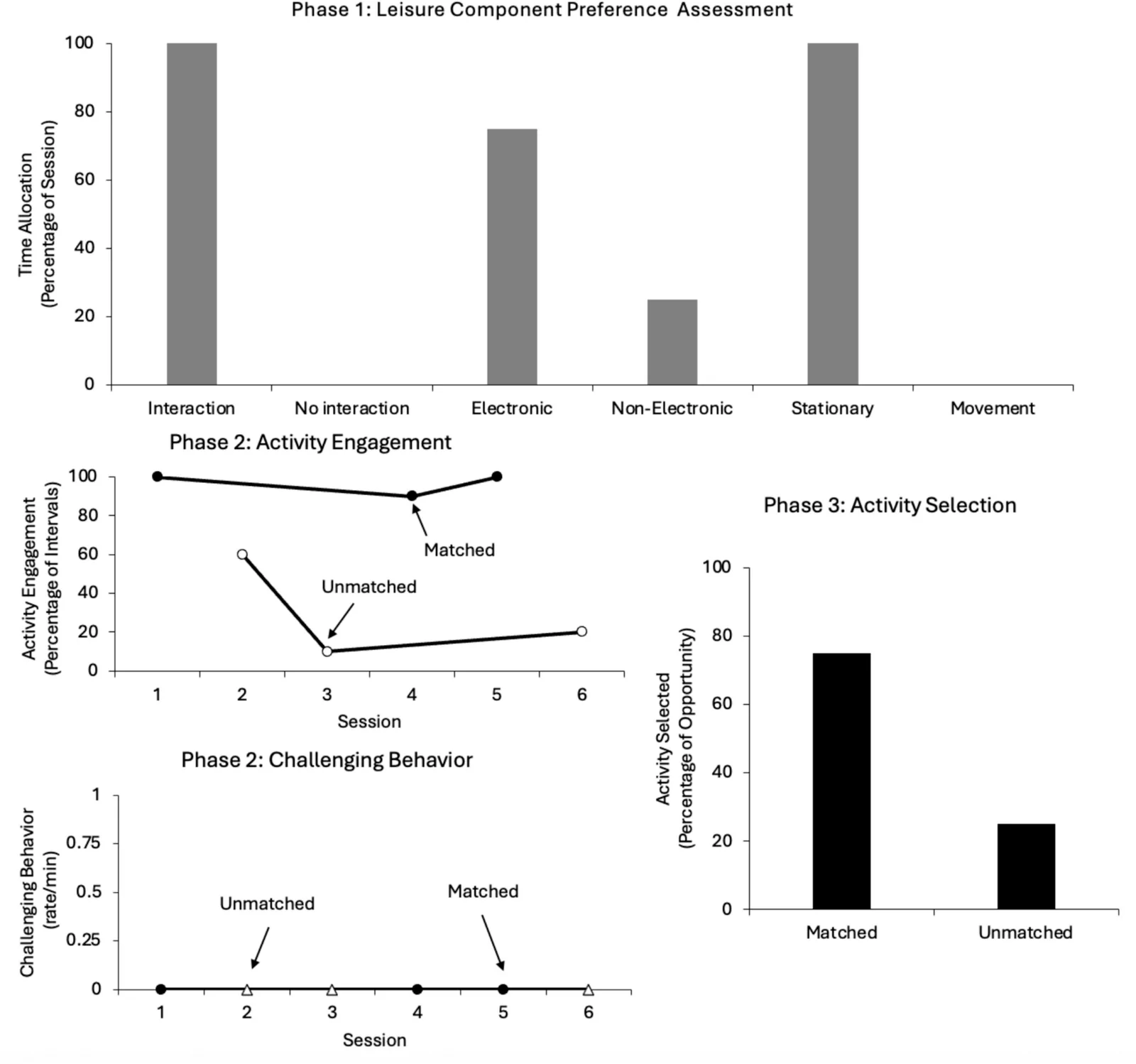
Results for Andrew. *Note*. The top panel depicts the leisure assessment results (Phase 1). The middle-left and bottom-left panels show activity engagement and challenging behavior comparisons from Phase 2. The fourth panel (bottom right) depicts the preference for matched and unmatched activities (Phase 3)

Figure 1 depicts Andrew’s results. Results of the leisure component preference assessment (Phase 1) are summarized in the top panel. The assessment results indicated that Andrew allocated 100% of the time to interaction, 100% of the time to stationary activities, and 75.0% of the time to electronic activities. On the basis of the assessment results, a matched profile involved interactive, sedentary, and electronic activities. An unmatched profile involved noninteractive, movement, and nonelectronic activities.

Results of the leisure profile analysis (Phase 2) are summarized in the middle and bottom left two panels of Fig. 1. Andrew’s activity engagement is depicted in the middle-left panel. Andrew engaged with the matched activity during 96.7% of 10-s intervals. He was engaged with the unmatched activity during 30.0% of 10-s intervals. Andrew’s combined inappropriate behavior is depicted in the bottom left of Fig. 1. Andrew did not engage in target behavior during matched or unmatched activities. Results of the activity preference assessment (Phase 3) are depicted in the bottom right panel of Fig. 1. Andrew chose matched activities 75.0% of the time during the preference assessment.

### Jameson

**Fig. 2:**
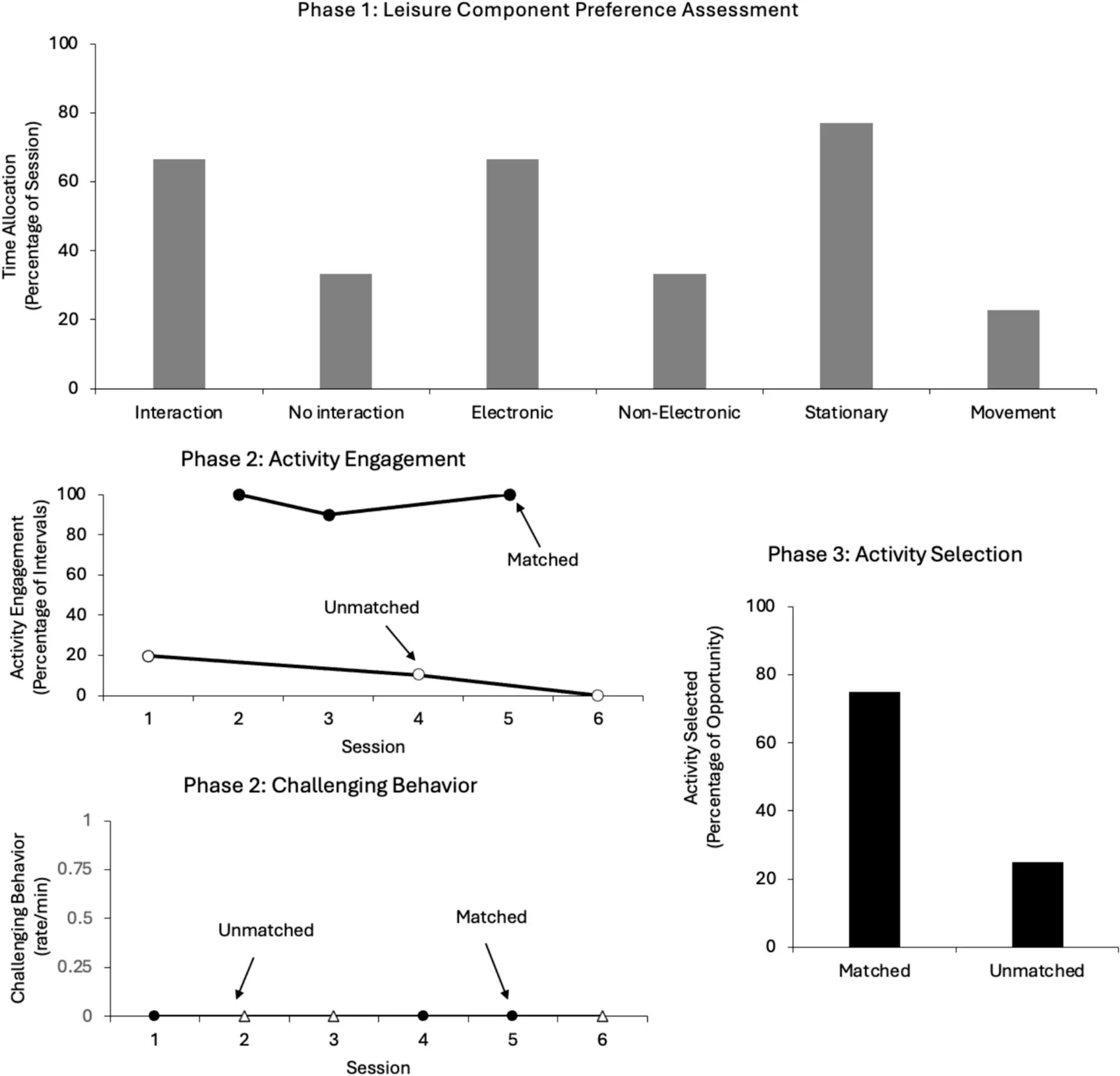
Results for Jameson. *Note.* The top panel depicts the leisure assessment results (Phase 1). The middle-left and bottom-left panels show activity engagement and challenging behavior comparisons from Phase 2. The fourth panel (bottom right) depicts the preference for matched and unmatched activities (Phase 3)

Figure 2 depicts Jameson’s results. Results of the leisure component preference assessment (Phase 1) are summarized in the top panel. The assessment results indicate that Jameson chose interaction 66.7% of the time, stationary activities 66.7% of the time, and electronic activities 77.7% of the time. On the basis of the assessment results, a matched profile involved interactive, sedentary, and electronic activities. An unmatched profile involved noninteractive, movement, and nonelectronic activities.

Results of the leisure profile analysis (Phase 2) are summarized in the middle and bottom left two panels of Fig. 2. Jameson’s activity engagement is depicted in the middle-left panel. Jameson engaged with the matched activity during 96.7% of 10-s intervals. He was engaged with the unmatched activity during 10.0% of 10-s intervals. Jameson’s combined inappropriate behavior is depicted in the bottom left panel of Fig. 2. Jameson engaged in no target behavior during matched and unmatched activities. The results of the activity preference assessment (Phase 3) are depicted in the bottom right of Fig. 2. Jameson chose matched activities 75.0% of the time during the preference assessment.

### Liam

**Fig. 3:**
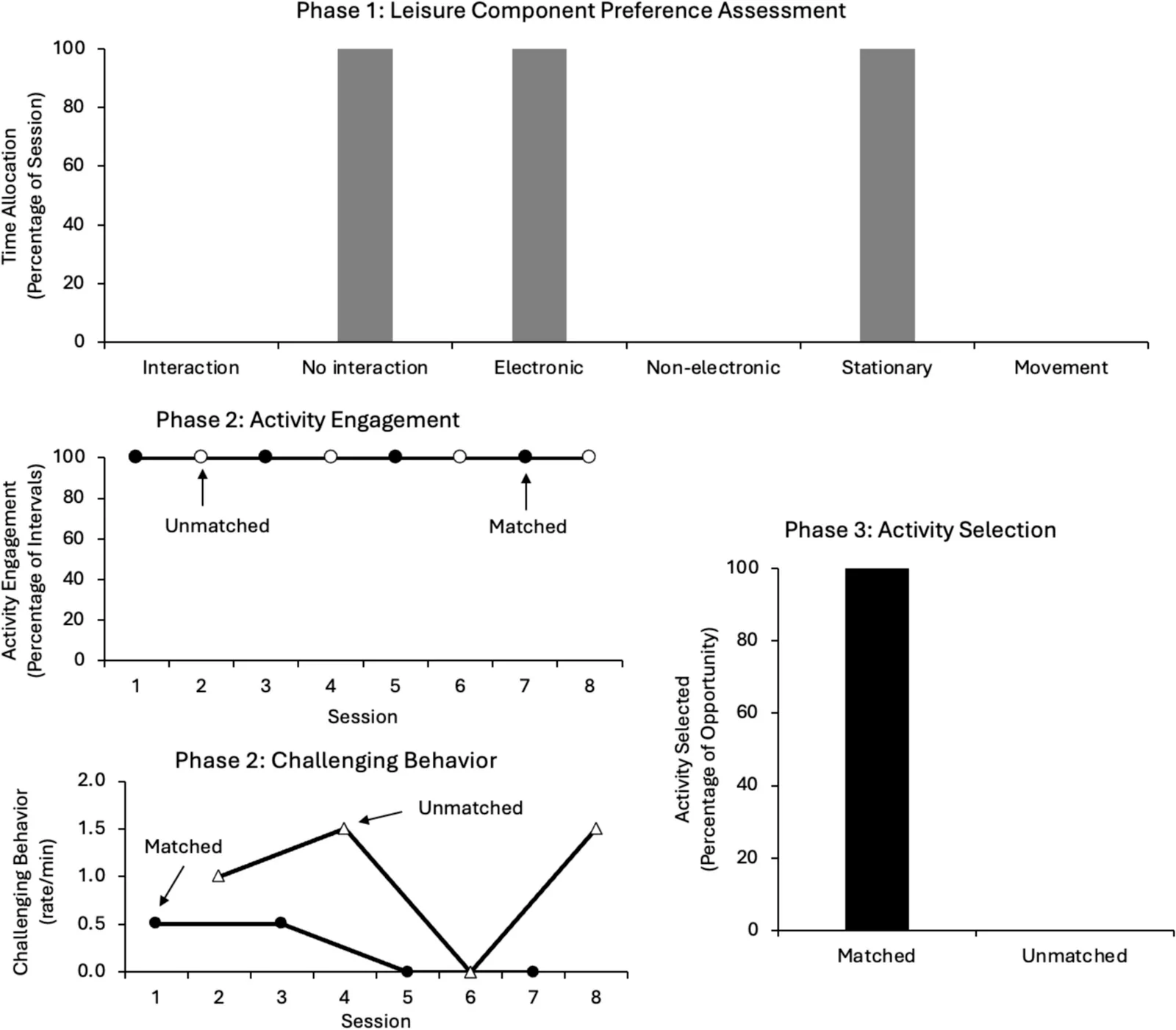
Results for Liam. *Note.* The top panel depicts the leisure assessment results (Phase 1). The middle-left and bottom-left panels show activity engagement and challenging behavior comparisons from Phase 2. The fourth panel (bottom right) depicts the preference for matched and unmatched activities (Phase 3)

Figure 3 depicts Liam’s results. Results of the leisure component preference assessment (Phase 1) are summarized in the top panel. The assessment results indicate that Liam chose no interaction 100% of the time, stationary activities 100% of the time, and electronic activities 100% of the time. On the basis of the assessment results, a matched profile was noninteractive, sedentary, and electronic activities. An unmatched profile was interactive, movement, and nonelectronic activities.

Results of the leisure profile analysis (Phase 2) are summarized in the middle and bottom left two panels of Fig. 3. Liam’s activity engagement is depicted in the middle-left panel. Liam engaged with matched and unmatched activities during 100% of 10-s intervals. Liam’s combined inappropriate behavior is depicted in the bottom left panel of Fig. 3. Liam engaged in inappropriate behavior at a rate of 1.0 per minute during unmatched activities but only 0.25 per minute during matched activities. Results of the activity preference assessment (Phase 3) are depicted in the bottom right panel of Fig. 3. Liam chose matched activities 100% of the time during the preference assessment.

## Discussion

The current study aimed to create a component-based assessment to match autistic adults with preferred leisure activities. Our primary goal was to pilot a direct assessment to predict which leisure activities three adults diagnosed with ASD might be more likely to engage with and prefer. This assessment allowed us to evaluate, validate, and incorporate client preferences into finding new leisure activities for three adults with high support needs.

In Phase 1, we used a concurrent-operant assessment to create a profile of preferred leisure activities for each participant. Participants showed clear preferences for each variable tested, although Jameson’s preferences were less pronounced than the others. All three participants preferred sedentary activities over those that required movement and electronic activities over those with a nonelectronic interface. Andrew and Jameson preferred interaction and Liam preferred no interaction. In Phase 2, we selected activities that matched or were unmatched to the Phase 1 profile. We found that participants had higher levels of engagement during matched activities than unmatched activities. Liam had lower rates of problem behavior when engaged in matched versus unmatched activities. In Phase 3, participants could choose between matched and unmatched activities. All participants preferred the matched activities.

Although numerous SPAs have been developed over the years, this study represents the first direct assessment explicitly designed to assess preference for and independent engagement with leisure experiences (Manente et al., 2022). While other SPA methods help identify specific items or activities an individual might like, the component-based model provides valuable information about how they interact with leisure activities. In addition, this methodology may allow researchers to efficiently sample, select, and eliminate many potential leisure activities, as activities are selected based on components or dimensions that span many individual activities. This method could be used in conjunction with traditional SPA methodology, with traditional SPAs identifying preferred items and the component-based assessment providing information about the context in which they would prefer to use them (e.g., with others, while seated, electronic format).

In the absence of evidence-based guidance regarding programming for autistic adults, adult programs focus their limited resources on employment outcomes and goals carried over from school years (e.g., academic or prevocational) while failing to develop leisure skills. However, there is much time each day when individuals are not working and should not be working. Participating in preferred leisure activities can make this time meaningful. As individuals enter the world of adult services, they encounter the *services cliff* (Roux et al., 2015). As a result, adult day programs are only funded for lean staffing ratios, which may create much downtime for clients. Therefore, the extent to which individuals can occupy themselves with activities they enjoy promotes meaningful activity while also potentially reducing the impact of behaviors that interfere with their well-being. Furthermore, although public education aims to prepare people for employment and community integration (IDEIA, 2004), individuals with autism need engaging activities to structure their downtime and live meaningful and fulfilling lives. Though there is room to improve employment outcomes for adults with ASD (Bush & Tassé, 2017), there is also room to improve the availability and quality of leisure activities, particularly for individuals with high support needs.

This study highlights the importance of incorporating choice and preference into the lives of individuals with ASD. In service of achieving habilitative goals, individuals with ASD are at risk of having their personal liberties (choices) limited or sacrificed by those who care for them. Behavior analysts must remain cognizant of individuals’ rights to balance their habilitative goals and engage in activities that may offer little to no direct benefit (Bannerman et al., 1990). As an example of the importance of this kind of assessment, one participant, Liam, was equally engaged during matched and unmatched activities; however, he still clearly preferred the matched leisure activities. Though it could be seen as beneficial that Liam was engaged even in non-preferred activities, the nature of those activities—involving another person—may have subtly compelled his participation. By more thoroughly understanding his preferences in this assessment, we can encourage his participation in activities that he, and not his staff, prefers. Measuring client preference allows participants to choose leisure activities they want to engage in. Unlike jobs matched to vocational profiles, engagement in matched leisure activities need not be limited by availability or for other practical reasons. 

All participants in the current study had moderate to severe intellectual disabilities. Conducting assessments with this population can be challenging from a methodological standpoint. For example, the results of concurrent operant assessments may not always capture actual preference. Instead, they may be subject to biased responding (e.g., preference for a side of the room or using verbal directions as prompts). Jameson’s response allocation showed greater variability compared to the other participants. Despite this, activities aligned with his profile were associated with higher engagement rates and greater preference. This suggests that even when performance is variable in Phase 1, it can still yield a valuable profile for Phases 2 and 3. That said, the assessment is flexible enough to be adapted to many individuals and circumstances, which should be examined in the future. The SPA literature suggests that animated GIFs can effectively be used in preference assessments (Morris & Vollmer, 2020). Individuals with lesser support needs may be able to use variations of this assessment that involve such GIFs presented concurrently instead of a whole room divided with tape.

The current study suggests a few additional directions for future research. The participants in this study were adults, and although increasing the evidence base for adults with ASD is valuable (Gerhardt & Lainer, 2011), this leisure assessment could be extended to a broader age range. Engaging in leisure activities is appropriate for individuals of all ages and levels of support. Additionally, there is a growing part of the adult population of individuals with ASD who have reached or are close to the traditional retirement age. This assessment may aid practitioners during transition planning to find engaging and preferred leisure activities to occupy clients’ time in meaningful ways despite age-related changes (e.g., no longer being able to stand for long periods or difficulty changing directions quickly).

Although participants displayed clear preferences for the three components assessed, these components were not meant to be exhaustive. Future extensions of this assessment should involve identifying other critical components of leisure activities that clients may prefer. In addition to the components of the activities themselves, examining environmental components, such as indoor versus outdoor activities, quiet versus noisy environments, and sparse versus crowded environments, could be considered. Future research should examine preference for matched leisure activities across similar environmental settings and situations.

As the current investigation was preliminary, several additional limitations could be incorporated into future research. Although the electronic versus nonelectronic component was equated by difficulty, greater care should be taken to equate the tasks in the movement versus stationary component. For example, if shooting a basketball in a hoop in a courtyard is the task in the movement component, then the task in the stationary component could be a seated basketball game, possibly table-top, to try to preserve the necessary features of the activity (e.g., get the ball in the hoop) with more and less movement. The procedures in the current investigation addressed this inconsistently. Similarly, the current study used a variety of individuals as communicative partners (ranging from novel to well-known) but did so based on convenience. In future research, the communicative partner could be more carefully controlled and evaluated across the novel, familiar, and well-known individuals in the client’s life. Furthermore, conversation could be constrained to specific topics, and the communicative partner could deliver verbal statements on a fixed- or variable-time schedule.

Additional limitations concern the generalizability and social validity of the current investigation. We observed each participant in Phase 2 for six to eight minutes with matched and unmatched activities. While we demonstrated engagement with activities for this period, it is possible that these results may not be generalized to the longer term and that individuals may become satiated with these activities. Should satiation arise, a recommendation for programming based on this assessment would be to identify several new leisure activities that match the learner’s profile and allow the client to choose among them. Although we found new leisure activities that the clients had not been observed to engage with at the center, the activities described in this study represent a relatively small sample of the overall range of possibilities. Future research should address this limitation to ensure the assessment can support other leisure activities. Social validity data from the clients’ staff and caregivers should be collected in future investigations to ensure that leisure activities are meaningful to all stakeholders. Collecting these data would also allow cultural variables and variations in play and leisure to be assessed. That said, the leisure activities in the current investigation were all based on those clients in the center currently engaged, indicating some degree of social validity for the setting.

Data on the occurrence of problem behavior were collected in Phase 2. Liam engaged in less problem behavior when a new leisure activity matched the profile generated in Phase 1. While we assessed preference for presumably enjoyable activities, encouraging engagement in any activity increases the risk of disruptive behavior (e.g., to escape). During the current study, we saw minimal problem behavior. However, this may have been because two participants had established behavior plans, and the third had no history of escape-maintained problem behavior. Future researchers should incorporate FA procedures into the assessment to better understand the relationship between leisure participation and behavioral function. Additionally, considerations of how challenging behavior may require modification to the procedure could be considered, as has been the case with other SPAs (Kang et al., 2010).

Future work could also measure behavioral indicators of happiness (Green & Reid, 1996). These indices help to assess the emotional state of individuals with profound disabilities who cannot communicate their feelings through traditional self-report methods. Comparisons could be made between matched and unmatched conditions on frequency and duration of happiness (e.g., smiling, laughing, and yelling while smiling) and unhappiness (e.g., frowning, grimacing, crying, and yelling without smiling). These supplemental dependent measures can help behavior analysts understand when novel activities are particularly preferred (or disliked) by clients and should increase the social validity of the assessment. Although this investigation was preliminary, future research should collect and report data on procedural fidelity.

There is a rich literature on teaching and increasing engagement with leisure skills to individuals with ASD and other developmental disabilities (Cannella-Malone et al., 2016; Roscoe et al., 2024). The results of component-based assessments might be used to identify new leisure skill targets for instruction. Future research could assess the predictive validity of this assessment in terms of which new leisure activities could be taught most efficiently. New leisure activities matched to profile may reach mastery faster and be more likely to be maintained than unmatched ones when taught using evidence-based methods. Additionally, all three participants in the current investigation preferred sedentary activities over those that require movement. Adolescents with ASDs tend to engage in lower levels of physical activity and have a higher prevalence of obesity compared to neurotypical peers (McCoy & Morgan, 2020). Future research may investigate whether exposure to activities requiring more physical movement or economic manipulations to promote the selection of more strenuous activities might shift clients’ preferences toward increased physical exercise (LaRue et al., 2023).

The current study successfully extended the work of LaRue et al. (2020) to leisure activities. This leisure activity assessment, LaRue and colleagues’ vocational assessment, and any other similar direct assessments could be coupled together, modified on the basis of support needs, and extended in numerous ways to support various leisure and employment-related goals and needs for individuals across the autism spectrum. These assessments could be conducted longitudinally several times to aid transition planning from school-based to adult services.

One of the hallmarks of behavior analysis is matching behavioral treatment to assessment results, where the assessments are based on data collection and direct observation. This assessment uses a direct approach to identify engaging leisure activities for adults with ASD who have substantial support needs. Although additional work is needed to support the use of this assessment, we were able to match each client in the assessment to two new leisure activities that were preferred and were engaged with spontaneously and independently.

## Data Availability

The author confirms that all data generated or analyzed during this study are included in this published article.
